# Site-selective radiolabeling using mushroom tyrosinase and the strain-promoted oxidation-controlled 1,2-quinone cycloaddition†

**DOI:** 10.1039/d3ra03486k

**Published:** 2023-06-12

**Authors:** Cindy Rodriguez, Samantha Delaney, Joni Sebastiano, Samantha M. Sarrett, Mike A. Cornejo, Sarah Thau, Meena M. Hosny, Brian M. Zeglis

**Affiliations:** a PhD Program in Chemistry, Graduate Center of the City University of New York New York New York 10016 USA bz102@hunter.cuny.edu; b Department of Chemistry, Hunter College, City University of New York New York New York 10065 USA; c Department of Radiology, Memorial Sloan Kettering Cancer Center New York New York 10021 USA; d PhD Program in Biochemistry, Graduate Center of the City University of New York New York New York 10016 USA; e Department of Radiology, Weill Cornell Medical College New York New York 10021 USA

## Abstract

We report the *in vitro* characterization and *in vivo* evaluation of a novel ^89^Zr-labeled radioimmunoconjugate synthesized using a site-selective bioconjugation strategy based on the oxidation of tyrosinase residues exposed by the deglycosylation of the IgG and the subsequent strain-promoted oxidation-controlled 1,2-quinone cycloaddition between these amino acids and *trans*-cyclooctene-bearing cargoes. More specifically, we site-selectively modified a variant of the A33 antigen-targeting antibody huA33 with the chelator desferrioxamine (DFO), thereby producing an immunoconjugate (DFO-^SPOCQ^huA33) with equivalent antigen binding affinity to its parent immunoglobulin but attenuated affinity for the FcγRI receptor. This construct was subsequently radiolabeled with [^89^Zr]Zr^4+^ to create a radioimmunoconjugate — [^89^Zr]Zr-DFO-^SPOCQ^huA33 — in high yield and specific activity that exhibited excellent *in vivo* behavior in two murine models of human colorectal carcinoma.

## Abstract 2

For the first time, mushroom tyrosinase and the strain-promoted oxidation-controlled 1,2-quinone (SPOCQ) cycloaddition have been harnessed for the site-selective bioconjugation of a radioimmunoconjugate.

## Graphical abstract

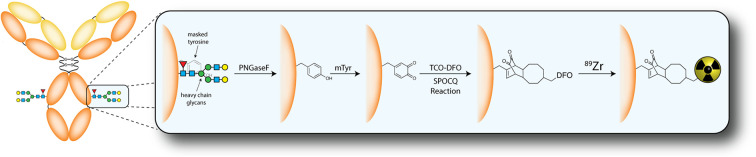

Monoclonal antibodies (mAb) have become indispensable tools in oncology for the delivery of a wide variety of cargoes to tumor tissue, including toxins, fluorophores, and radionuclides for both imaging and radioimmunotherapy.^1^ Traditionally, these payloads have been attached to mAbs *via* the stochastic modification of lysine residues within the immunoglobulins.^2^ This approach is admittedly simple, but it risks impairing the immunoreactivity of the mAb if the complementarity determining regions are inadvertently perturbed, and it inevitably produces a poorly defined, heterogeneous, and complex mixture of regioisomeric products.^3^

To circumvent these issues, a great deal of work has been dedicated to the development of site-specific and site-selective approaches to bioconjugation.^4,5^ Several effective and elegant methodologies have been created, including strategies based on the modification of thiols, the manipulation of the heavy chain glycans, and the incorporation of unnatural amino acids and peptide tags into immunoglobulins. A significant body of preclinical research has demonstrated that site-specifically and site-selectively modified immunoconjugates outperform their stochastically labeled cousins both *in vitro* and *in vivo*.^6–8^ Yet the development of site-selective bioconjugation approaches that balance selectivity, stability, and ease has proven challenging. Indeed, each strategy has its own set of limitations. For example, the selective modification of cysteine residues with maleimide-bearing probes is facile, but the linkage between these two moieties is unstable *in vivo*.^9^ On the other hand, the use of click chemistry to modify unnatural amino acids is both specific and stable, but the genetic engineering needed to generate these immunoglobulins is expensive and complex.^10^ Finally, chemoenzymatic approaches to the modification of the heavy chain glycans offer exquisite specificity, stability, and modularity, but they can require expensive reagents, and the use of novel enzymes can complicate clinical translation.

Here, we describe the synthesis of a ^89^Zr-labeled radioimmunoconjugate using an emergent chemoenzymatic approach to site-selective bioconjugation. This strategy is predicated on (i) the removal of the IgG's heavy chain glycans with PNGaseF to expose a quartet of solvent-accessible tyrosine residues, (ii) the oxidation of these tyrosines to 1,2-orthoquinones with mushroom tyrosinase (mTyr), and (iii) the modification of these orthoquinones with *trans*-cyclooctene (TCO)-bearing cargoes *via* the strain-promoted oxidation-controlled 1,2-quinone (SPOCQ) cycloaddition (Schemes 1 and 2).^11^

**Scheme 1:**
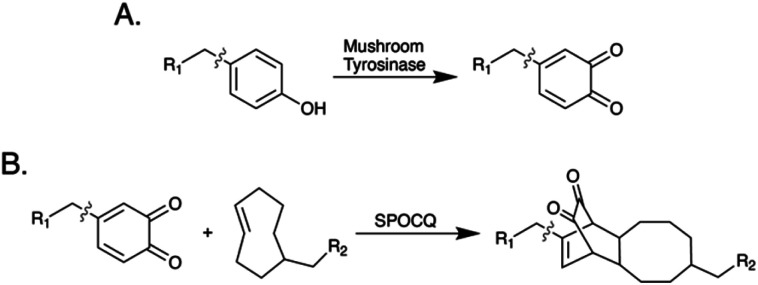
(A) The oxidation of tyrosine by mushroom tyrosinase; (B) the strain-promoted oxidation-controlled 1,2-quinone cycloaddition.

While the synthesis of ADCs using this bioconjugation strategy has previously been reported, this work represents the first application of this technology to radiopharmaceutical chemistry and — more importantly — the first *in vivo* evaluation of any immunoconjugate synthesized in this manner.^12–14^ Indeed, we contend that this strategy represents a step forward compared to extant chemoenzymatic approaches to radiolabeling because it (a) is faster, (b) involves fewer steps, (c) relies on cheaper and easier-to-make reagents, and (d) employs more widely used and commonly available enzymes.

The model system assembled for this investigation was composed of (1) the huA33 antibody, an mAb that targets the A33 antigen expressed on >95% of colorectal cancers; (2) ^89^Zr, a positron-emitting radiometal whose 3.3 d physical half-life aligns well with the multi-day serum residence times of IgGs; (3) a TCO-bearing variant of desferrioxamine (TCO-DFO), an acyclic chelator that provides Zr^4+^ with an oxygen-rich, kinetically inert, and thermodynamically stable coordination environment; and (4) the A33 antigen-expressing SW1222 human colorectal cancer cell line. Before embarking on the synthesis of a ^89^Zr-labeled mAb, however, we first validated our procedures using a TCO-bearing derivative of the near-infrared fluorophore Cy5 (TCO-Cy5). The first step in the bioconjugation procedure is the deglycosylation of the mAb to expose a quartet of tyrosine residues at positions Y296 and Y300 that are normally masked by the heavy chain glycans (Scheme 2).^15^ To this end, native huA33 was deglycosylated *via* incubation with PNGaseF for 6 h at 37 °C and subsequently purified using magnetic chitin beads to yield ^degly^huA33. This intermediate was then modified with TCO-Cy5 in a one-pot procedure in which ^degly^huA33 was incubated in PBS (pH 5.5) overnight at 4 °C with 12 equiv. of mTyr as well as 10 equiv. of the TCO-bearing fluorophore. This reaction mixture was ultimately purified using protein A chromatography to yield the final product: Cy5-^SPOCQ^huA33.

**Scheme 2:**
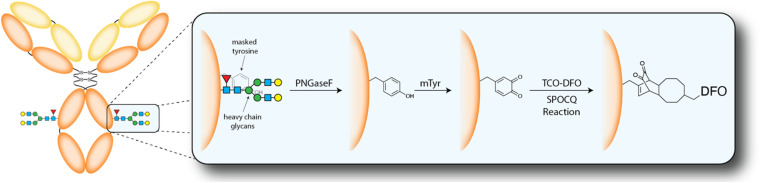
Schematic of the deglycosylation of an antibody followed by the mTyr- and SPOCQ cycloaddition-mediated modification of these residues with TCO-DFO.

SDS-PAGE helped illustrate the importance of each of the methodology's three ingredients: PNGaseF, mTyr, and TCO-Cy5 (Fig. 1). The treatment of wild-type huA33 (Lane 1) with PNGaseF clearly selectively reduces the molecular weight of the heavy chain (Lane 4), and the subsequent incubation of this ^degly^huA33 product with mTyr and Cy5-TCO yields an immunoconjugate — Cy5-^SPOCQ^huA33 — bearing fluorophores appended only to the heavy chain (Lane 6). Critically, labeling huA33 with TCO-Cy5 alone (Lane 2) or TCO-Cy5 + PNGaseF (Lane 5) did not produce a fluorophore-bearing immunoconjugate. Interestingly, however, the treatment of huA33 with mTyr + TCO-Cy5 did result in a very low degree of conjugation (Lane 3), suggesting that (a) Y296 and Y300 are slightly accessible when the glycans are still attached or (b) mTyr and TCO-Cy5 are inefficiently labeling another tyrosine residue within the heavy chain.

**Fig. 1:**
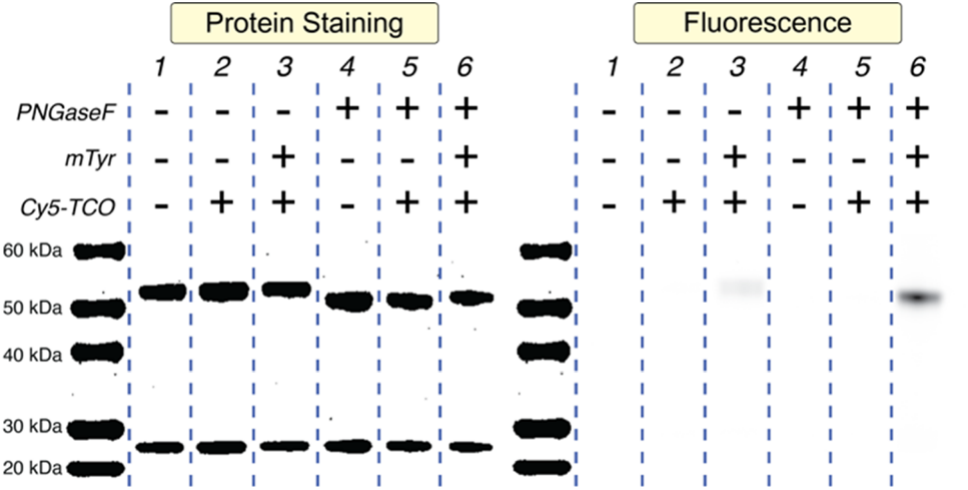
Protein-stained and fluorescent SDS-PAGE gel electrophoresis of huA33 in various reaction conditions.

UV-vis spectrophotometry revealed that Cy5-^SPOCQ^huA33 had a degree of labeling of ∼1.9 ± 0.0 Cy5 per mAb. This value — despite the excess of mTyr and Cy5-TCO — suggests either that one of the two tyrosines on each heavy chain is preferentially modified or that the modification of one tyrosine prevents the modification of the other. Efforts to speed up the methodology were largely unsuccessful: PNGaseF treatment <6 h resulted in incomplete deglycosylation; shorter mTyr + Cy5-TCO incubations could only provide comparable DOLs with much higher concentrations of reagents; and a three-reagents-one-pot approach proved untenable due to buffer incompatibilities (Fig. S1 and Table S1†). Finally, to illustrate the modularity of this approach, this strategy was successfully employed to attach Cy5 to two other IgG_1_—the HER2-targeting mAbs pertuzumab and trastuzumab—ultimately producing immunoconjugates with DOLs of 1.7 ± 0.2 and 1.6 ± 0.2, respectively (Fig. S2, S3 and Table S2†).

With the initial optimization of the system complete, we next turned to the use of this methodology for the synthesis of ^89^Zr-labeled radioimmunoconjugates. To this end, TCO-DFO was synthesized *via* the incubation of *p*-SCN-Bn-DFO with 5 equiv. of an amine-bearing variant of TCO and 2 equiv. of DIPEA in DMSO for 1 h at 25 °C, purified *via* preparative HPLC, and characterized *via* ESI-MS (Fig. S4†). Subsequently, huA33 was treated with PNGaseF to produce ^degly^huA33, and the deglycosylated mAb was treated overnight at 4 °C with 10 equiv. of TCO-DFO and 12 equiv. of mTyr to yield DFO-^SPOCQ^huA33 (Scheme 2). To provide a point of comparison, a stochastically modified immunoconjugate — DFO-huA33 — was synthesized *via* the reaction of huA33 with 20 equiv. of *p*-SCN-Bn-DFO in PBS (pH 8.8–9.0) at 37 °C for 1 h and purified using size exclusion chromatography. MALDI-ToF analysis revealed that the DOLs of DFO-^SPOCQ^huA33 and DFO-huA33 were 1.6 ± 0.1 and 1.7 ± 0.1 DFO per mAb, respectively (Table S3†).

The *in vitro* characterization of DFO-^SPOCQ^huA33 and DFO-huA33 revealed several key similarities and differences between the two immunoconjugates. An ELISA assay with recombinant A33 antigen demonstrated that native huA33, ^degly^huA33, DFO-huA33, and DFO-^SPOCQ^huA33 all bind their target antigen with comparable affinity (Fig. 2A). However, an ELISA with FcγRI underscored a substantial difference between the behavior of the two probes. FcγRI is an Fcγ receptor capable of binding monomeric immunoglobulins that is expressed by monocytes, macrophages, and tissue-resident macrophages in organs like the liver and spleen. In this case, the two deglycosylated immunoconjugates — ^degly^huA33 and DFO-^SPOCQ^huA33 — exhibited dramatically reduced binding to FcγRI compared to the fully glycosylated wild-type huA33 and DFO-huA33 (Fig. 2B). This observation aligns with literature findings that have demonstrated that the removal or truncation of the heavy chain glycans prompts a conformational shift in the C_H_2 domain of IgGs that interferes with the receptor's binding site. Our own lab has previously explored this phenomenon in the context of deglycosylated ^89^Zr-labeled mAbs (*vide infra*).^6,16–18^

**Fig. 2:**
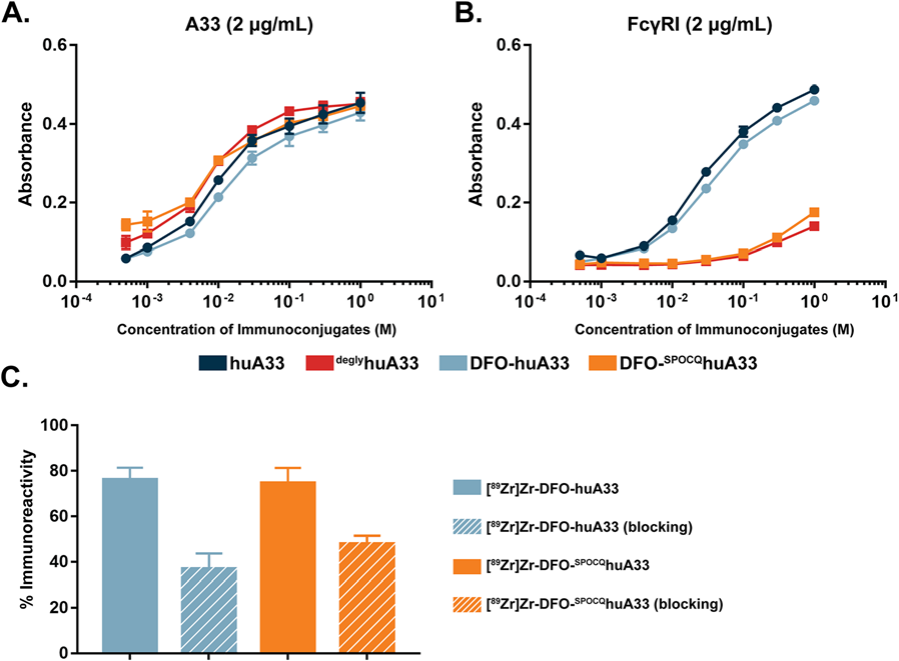
ELISA assays exploring the binding of huA33, ^PNGaseF^huA33, DFO-huA33, and DFO-^SPOCQ^huA33 with (A) the A33 antigen and (B) FcγRI as well as (C) a cell-based immunoreactivity assay of [^89^Zr]Zr-DFO-huA33 and [^89^Zr]Zr-DFO-^SPOCQ^huA33 with A33 antigen-expressing SW1222 colorectal cancer cells (*n* = 3).

The two DFO-bearing immunoconjugates were radiolabeled with [^89^Zr]Zr^4+^ using standard protocols to produce [^89^Zr]Zr-DFO-huA33 or [^89^Zr]Zr-DFO-^SPOCQ^huA33 in >95% radiochemical yield and specific activities of 4.9 ± 0.3 mCi mg^−1^ and 4.9 ± 0.2 mCi mg^−1^, respectively (Fig. S5†). Autoradiographic SDS-PAGE of the radioimmunoconjugates helped illustrate the site-selectivity of the conjugation in which activity was detected on both the heavy- and light-chains of [^89^Zr]Zr-DFO-huA33 while only on the heavy-chains of [^89^Zr]Zr-DFO-^SPOCQ^huA33 (Fig. S6†). The stability of the radioimmunoconjugates was assayed *via* incubation in human serum at 37 °C for 5 days followed by radio-iTLC and radio-SE-HPLC. Both analytical methods revealed that the radioimmunoconjugates were >95% stable to demetallation and aggregation over this period (Fig. S7 and S8†). To conclude the *in vitro* characterization, A33 antigen-expressing SW1222 human colorectal cancer cells were employed to illustrate that both [^89^Zr]Zr-DFO-huA33 and [^89^Zr]Zr-DFO-^SPOCQ^huA33 demonstrated blockable binding and boasted immunoreactive fractions of >0.75 (Fig. 2C).

The final step in the validation of this SPOCQ cycloaddition-based approach to site-selective radiolabeling was the evaluation of the *in vivo* performance of [^89^Zr]Zr-DFO-^SPOCQ^huA33 in murine models of colorectal cancer. To this end, athymic nude mice bearing subcutaneous SW1222 colorectal carcinoma xenografts were intravenously injected with either [^89^Zr]Zr-DFO-huA33 or [^89^Zr]Zr-DFO-^SPOCQ^huA33 (100–110 μCi in 100 μL sterile PBS), and PET scans were collected 24, 72, and 120 h post-injection (Fig. 3). Both radiotracers demonstrated excellent *in vivo* performance. They effectively delineated tumor tissue as early as 24 h post-injection, with tumoral activity concentrations and tumor-to-background contrast reaching maxima at 120 h p.i. A biodistribution analysis performed after the final imaging timepoint reinforced the similar behavior of the two radioimmunoconjugates, as the [^89^Zr]Zr-DFO-^SPOCQ^huA33 and [^89^Zr]Zr-DFO-huA33 yielded comparable activity concentrations in the tumor (109 ± 17 and 70 ± 33 %ID g^−1^), blood (3.0 ± 0.8 and 2.1 ± 1.6 %ID g^−1^), liver (2.7 ± 0.8 and 4.0 ± 1.4 %ID g^−1^), and spleen (2.5 ± 1.1 and 2.0 ± 0.8 %ID g^−1^) at 120 h post-injection (Table S4†). While [^89^Zr]Zr-DFO-^SPOCQ^huA33 admittedly did not outperform [^89^Zr]Zr-DFO-huA33 in this model, it is important to note that huA33 is a highly optimized mAb and that the benefits of site-selective bioconjugation are likely to be more pronounced with less optimized mAbs. This case notwithstanding, the literature clearly underscores the advantages of site-selective and site-specific bioconjugation for radioimmunoconjugates.^6–8,16,19^

**Fig. 3:**
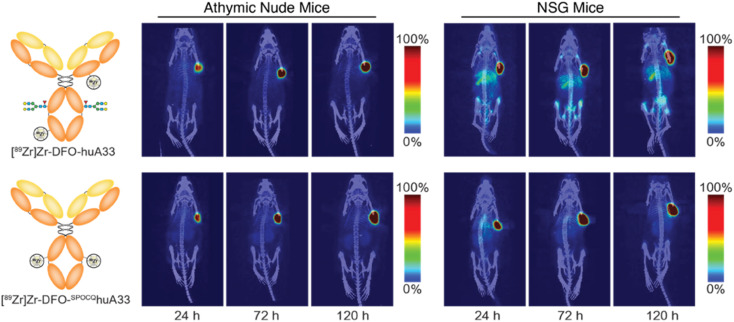
*In vivo* evaluation of the radioimmunoconjugates. Representative maximum intensity projection PET scans collected 24, 72, and 120 h after the intravenous administration of [^89^Zr]Zr-DFO-huA33 or [^89^Zr]Zr-DFO-^SPOCQ^huA33 [3.7–4.0 MBq (100–110 μCi), 20–22 μg, in 100 μL of PBS] to athymic nude or NSG mice bearing subcutaneous A33-expressing SW1222 colorectal cancer xenografts (*n* = 4).

Similar experiments in NSG mice bearing subcutaneous SW1222 xenografts produced strikingly different results (Fig. 3). In this case, the PET and biodistribution experiments revealed that [^89^Zr]Zr-DFO-^SPOCQ^huA33 produced higher activity concentrations in the tumor than [^89^Zr]Zr-DFO-huA33 (76 ± 13 *vs.* 33 ± 10 %ID g^−1^ at 120 h p.i.) as well as lower activity concentrations in the liver (5.9 ± 2.1 *vs.* 16 ± 0.6 %ID g^−1^) and spleen (9.7 ± 4.0 *vs.* 42 ± 8.6 %ID g^−1^) at the same timepoint (Table S5†). The most likely explanation for this phenomenon is that NSG mice (which lack T and B cells) do not produce endogenous mAbs, while athymic mice (which lack T cells but have B cells) do produce endogenous mAbs.^19^ As a result, the FcγRI of NSG mice are unoccupied and are thus able to bind and sequester [^89^Zr]Zr-DFO-huA33 (but not [^89^Zr]Zr-DFO-^SPOCQ^huA33) in the spleen and liver. The FcγRI of athymic mice, in contrast, are largely occupied by endogenous mAbs; therefore, they do not engage either radioimmunoconjugate, and neither tracer is preferentially sequestered in FcγRI-rich tissues. While the reduced FcγRI engagement of [^89^Zr]Zr-DFO-^SPOCQ^huA33 could improve clinical imaging (especially in the immunocompromised), it remains unclear whether this effect will provide benefits in patients.

In conclusion, this investigation represents the first use of a chemoenzymatic strategy based on PNGaseF, mTyr, and the SPOCQ ligation for the synthesis of a site-selectively modified radioimmunoconjugate as well as the first time that any mAb modified using this methodology has been evaluated in mice. In practice, this approach yielded a well-defined and homogeneous probe — [^89^Zr]Zr-DFO-^SPOCQ^huA33 — with high stability, high specific activity, and excellent *in vitro* and *in vivo* behavior. Indeed, the *in vivo* performance of [^89^Zr]Zr-DFO-^SPOCQ^huA33 matched that of a stochastically labeled analog ([^89^Zr]Zr-DFO-huA33) in an athymic mouse model of colorectal carcinoma and surpassed that of the randomly modified probe in an NSG mouse model of the disease. Going forward, we plan to further explore adapting this protocol into a one-pot procedure, probe the use of mTyr and the SPOCQ reaction for the radiolabeling of peptides, and investigate the creation of GMP-grade components of this strategy for the clinical production of radioimmunoconjugates.

## Ethical statement

All animal procedures were performed in accordance with the guidelines for the care and use of laboratory animals set forth by Weill Cornell Medical College and Hunter College and approved by the institutional animal care and use committees of both institutions.

## Author contributions

C. R. (investigation, conceptualization, writing original draft), S. D., J. S., S. M. S., M. C., S. T., M. M. H. (investigation), B. M. Z. (supervision, conceptualization, project administration, writing original draft, reviewing, and editing).

## Conflicts of interest

The authors declare no conflict of interest.

## Supplementary Material

RA-013-D3RA03486K-s001

## References

[cit1] Lin M., Paolillo V., Le D. B., Macapinlac H., Ravizzini G. C. (2021). Curr. Probl. Cancer.

[cit2] Agarwal P., Bertozzi C. R. (2015). Bioconjugate Chem..

[cit3] Wang L., Amphlett G., Blättler W. A., Lambert J. M., Zhang W. (2005). Protein Sci..

[cit4] Adumeau P., Sharma S. K., Brent C., Zeglis B. M. (2016). Mol. Imaging Biol..

[cit5] Adumeau P., Sharma S. K., Brent C., Zeglis B. M. (2016). Mol. Imaging Biol..

[cit6] Vivier D., Fung K., Rodriguez C., Adumeau P., Ulaner G. A., Lewis J. S., Sharma S. K., Zeglis B. M. (2020). Theranostics.

[cit7] Bai C., Reid E. E., Wilhelm A., Shizuka M., Maloney E. K., Laleau R., Harvey L., Archer K. E., Vitharana D., Adams S., Kovtun Y., Miller M. L., Chari R., Keating T. A., Yoder N. C. (2020). Bioconjugate Chem..

[cit8] Kristensen L. K., Christensen C., Jensen M. M., Agnew B. J., Schjöth-Frydendahl C., Kjaer A., Nielsen C. H. (2019). Theranostics.

[cit9] Shen B. Q. (2012). et al.. Nat. Biotechnol..

[cit10] Ahn S. H., Vaughn B. A., Solis W. A., Lupher Jr M. L., Hallam T. J., Boros E. (2020). Bioconjugate Chem..

[cit11] Borrmann A., Fatunsin O., Dommerholt J., Jonker A. M., Löwik D. W. P. M., van Hest J. C. M., van Delft F. L. (2015). Bioconjugate Chem..

[cit12] Bruins J. J., Damen J. A. M., Wijdeven M. A., Lelieveldt L. P. W. M., van Delft F. L., Albada B. (2021). Bioconjugate Chem..

[cit13] Cao W., Maza J. C., Chernyak N., Flygare J. A., Krska S. W., Toste F. D., Francis M. B. (2023). Bioconjugate Chem..

[cit14] Marmelstein A. M., Lobba M. J., Mogilevsky C. S., Maza J. C., Brauer D. D., Francis M. B. (2020). J. Am. Chem. Soc..

[cit15] Subedi G. P., Barb A. W. (2015). Structure.

[cit16] Vivier D., Sharma S. K., Adumeau P., Rodriguez C., Fung K., Zeglis B. M. (2019). J. Nucl. Med..

[cit17] Geuijen K. P. M., Oppers-Tiemissen C., Egging D. F., Simons P. J., Boon L., Schasfoort R. B. M., Eppink M. H. M. (2017). FEBS Open Bio.

[cit18] Radaev S., Sun P. D. (2001). J. Biol. Chem..

[cit19] Sharma S. K., Pourat J., Abdel-Atti D., Carlin S. D., Piersigilli A., Bankovich A. J., Gardner E. E., Hamdy O., Isse K., Bheddah S., Sandoval J., Cunanan K. M., Johansen E. B., Allaj V., Sisodiya V., Liu D., Zeglis B. M., Rudin C. M., Dylla S. J., Poirier J. T., Lewis J. S. (2017). Cancer Res..

