# Supplementary material for: Site-selective radiolabeling using mushroom tyrosinase and the strain-promoted oxidation-controlled 1,2-quinone cycloaddition
Source: RSC Adv. 2023 Jun 12;13(26):17705–9. doi: 10.1039/d3ra03486k (PMC10258682; doi:10.1039/d3ra03486k)
Supplement: RA-013-D3RA03486K-s001 [file RA-013-D3RA03486K-s001.pdf]

Electronic Supplementary Material (ESI) for RSC Advances.
This journal is © The Royal Society of Chemistry 2023

Supporting Information

Site-Specific Radiolabeling Using Mushroom Tyrosinase and the Strain-Promoted Oxidation-Controlled 1,2-Quinone Cycloaddition

Cindy Rodriguez[a,b,c], Samantha Delaney [b,c,d], Joni Sebastiano[b,c,d], Samantha M. Sarrett[b,c,d], Mike A. Cornejo[a,b,c], Sarah Thau[b], Meena M. Hosny[b], and Brian M. Zeglis[a,b,c,d,e]

[a]*Ph.D. Program in Chemistry, Graduate Center of the City University of New York, New York, New York 10016, United States*

[b]*Department of Chemistry, Hunter College, City University of New York, New York, New York, 10065 United States*

[c]*Department of Radiology, Memorial Sloan Kettering Cancer Center, New York, New York 10021 United States*

[d]*Ph.D. Program in Biochemistry, Graduate Center of the City University of New York, New York, New York 10016 United States*

[e]*Department of Radiology, Weill Cornell Medical College, New York, New York 10021 United States*

Corresponding Author: Brian M. Zeglis; 413 East 69th Street, New York, NY, 10021; Phone: 212-896-0433; E-mail: bz102@hunter.cuny.edu

Running Title: Site-Specific Radiolabeling Using Mushroom Tyrosinase and the Strain-Promoted Oxidation-Controlled 1,2-Quinone Cycloaddition

Keywords: Site-specific bioconjugation; site-selective bioconjugation; biorthogonal chemistry; click chemistry; immunoPET

SUPPLEMENTAL FIGURES

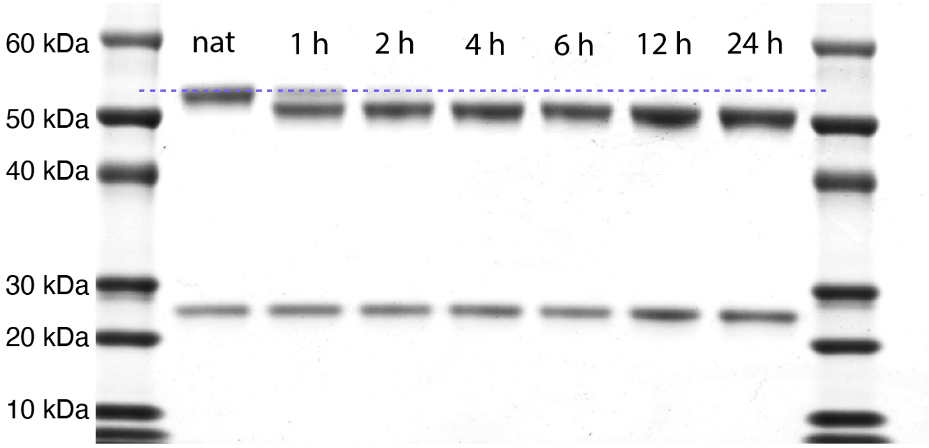

Figure S1. Protein-stained SDS-PAGE of huA33 exposed to PNGaseF for various amounts of time. The dotted line denotes the mass of the heavy chain of the parental IgG.

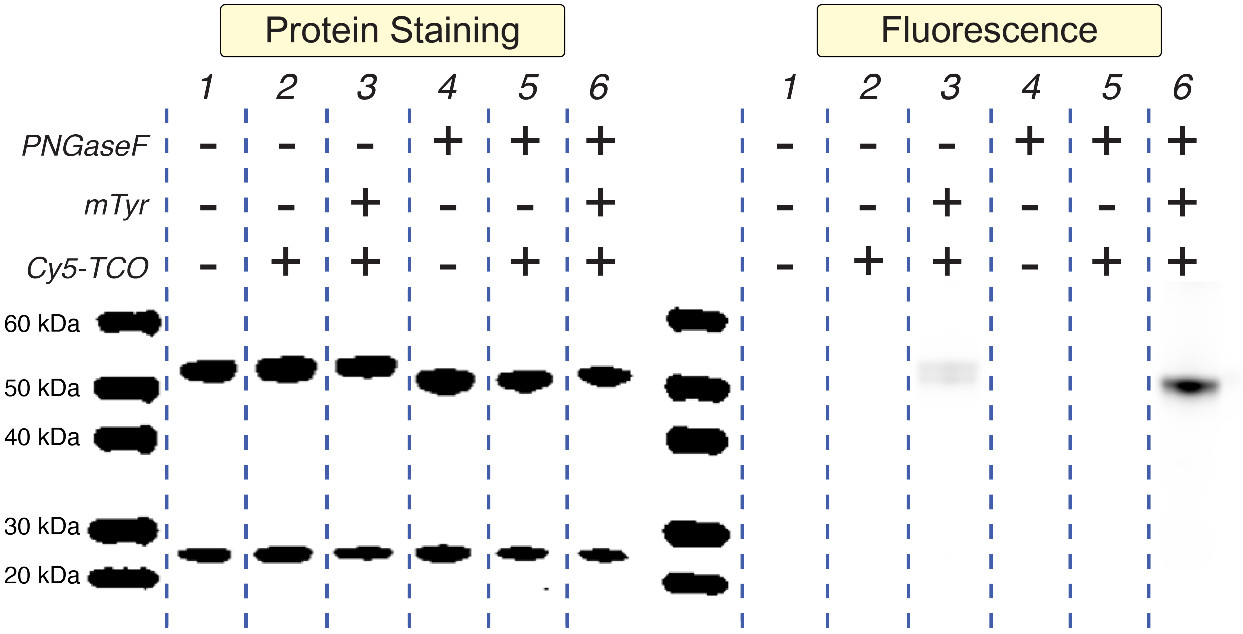

Figure S2. Protein-stained and fluorescent SDS-PAGE of pertuzumab modified under various conditions.

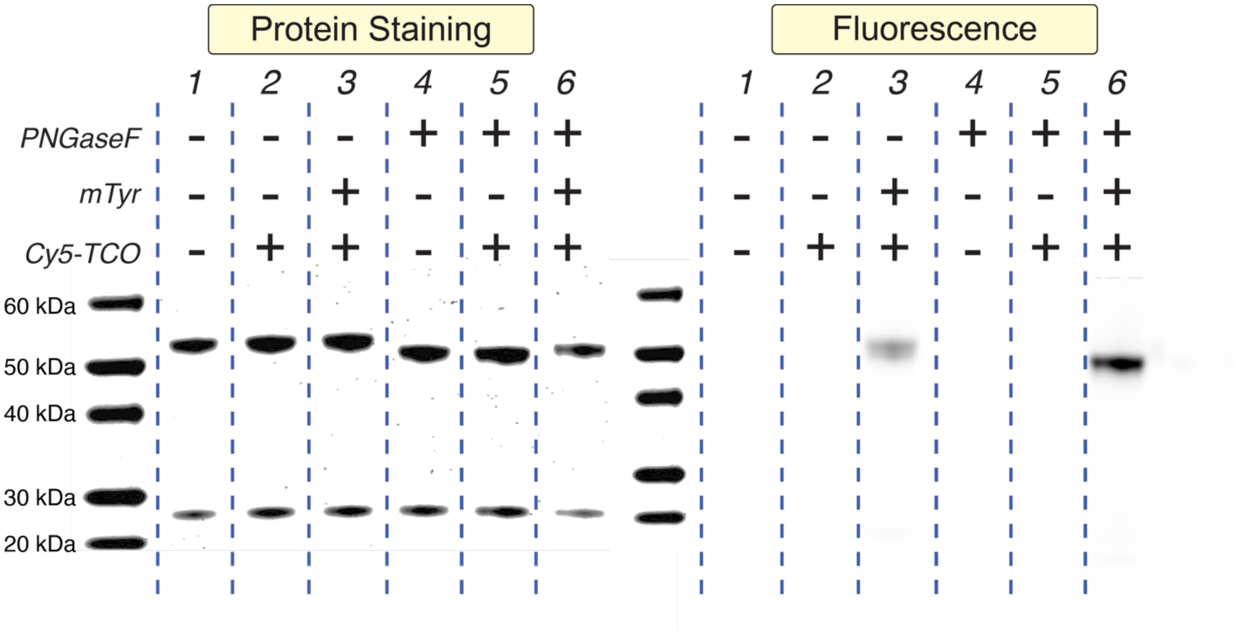

Figure S3. Protein-stained and fluorescent SDS-PAGE of trastuzumab modified under various conditions.

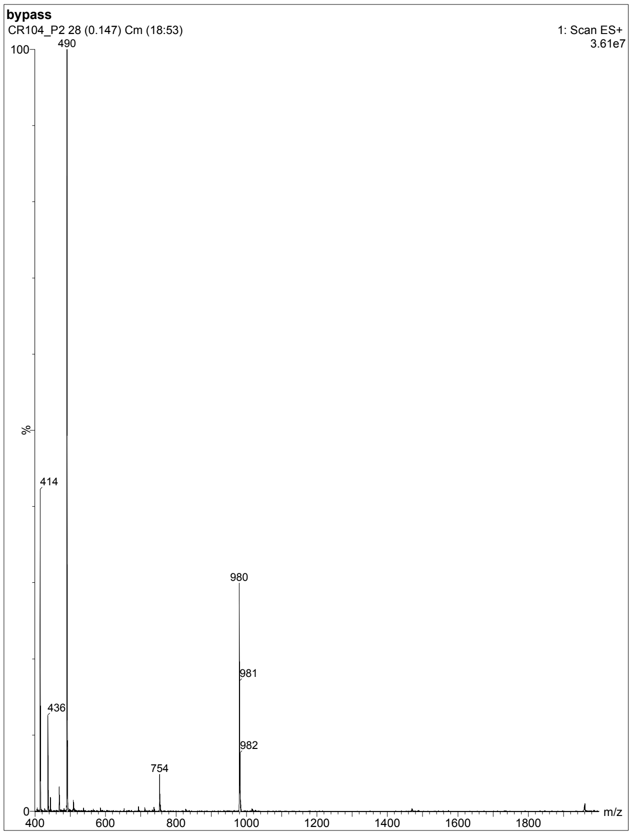

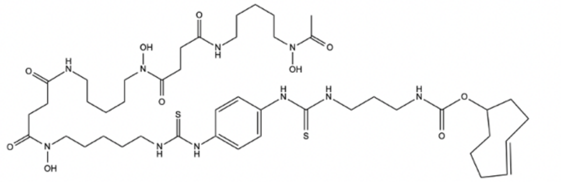

Chemical Formula: $C_{45}H_{74}N_{10}O_{10}S_2$
Exact Mass: 978.50
Molecular Weight: 979.27
m/z: 978.50 (100.0%), 979.51 (49.9%), 980.51 (15.1%), 980.50 (11.0%), 979.50 (5.3%), 981.50 (5.0%), 981.51 (3.6%), 982.51 (1.5%)
Elemental Analysis: C, 55.19; H, 7.62; N, 14.30; O, 16.34; S, 6.55

Figure S4. ESI-MS of purified DFO-TCO.

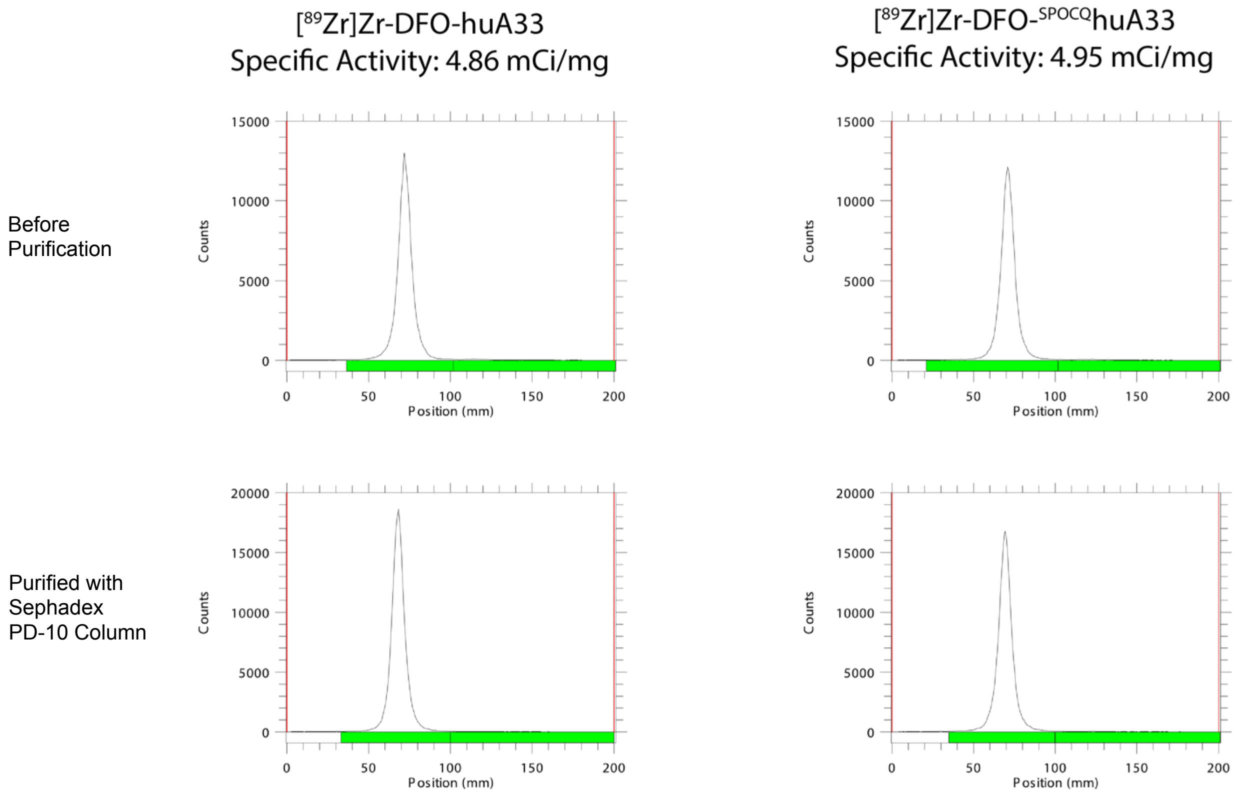

Figure S5. Representative radio-iTLC chromatograms. The radiolabeling of [^{89}Zr]Zr-DFO-huA33 and [^{89}Zr]Zr-DFO-^{SPOCQ}huA33 was followed via radio-iTLC using EDTA (50 mM, pH 5.0) as the eluent. Both constructs typically displayed >98% radiochemical purity both before and after purification via gel filtration. The peak at ~75 mm corresponds to the radiolabeled antibody; any free [^{89}Zr]Zr-EDTA would be seen at ~150 mm. All radiolabeling studies and iTLC scans were performed in triplicate.

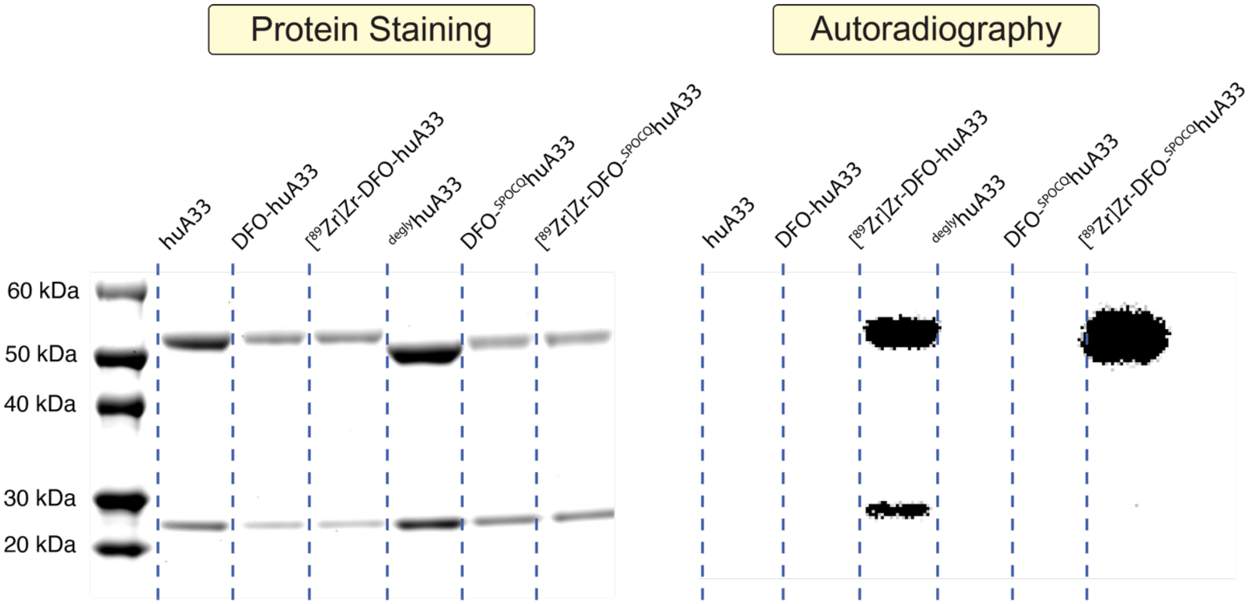

Figure S6. SDS-PAGE analysis of [^{89}Zr]Zr-DFO-huA33 and [^{89}Zr]Zr-DFO-^{SPOCQ}huA33. Protein-stained reducing SDS-PAGE of the radioimmunoconjugates as well as autoradiography of the stained gel.

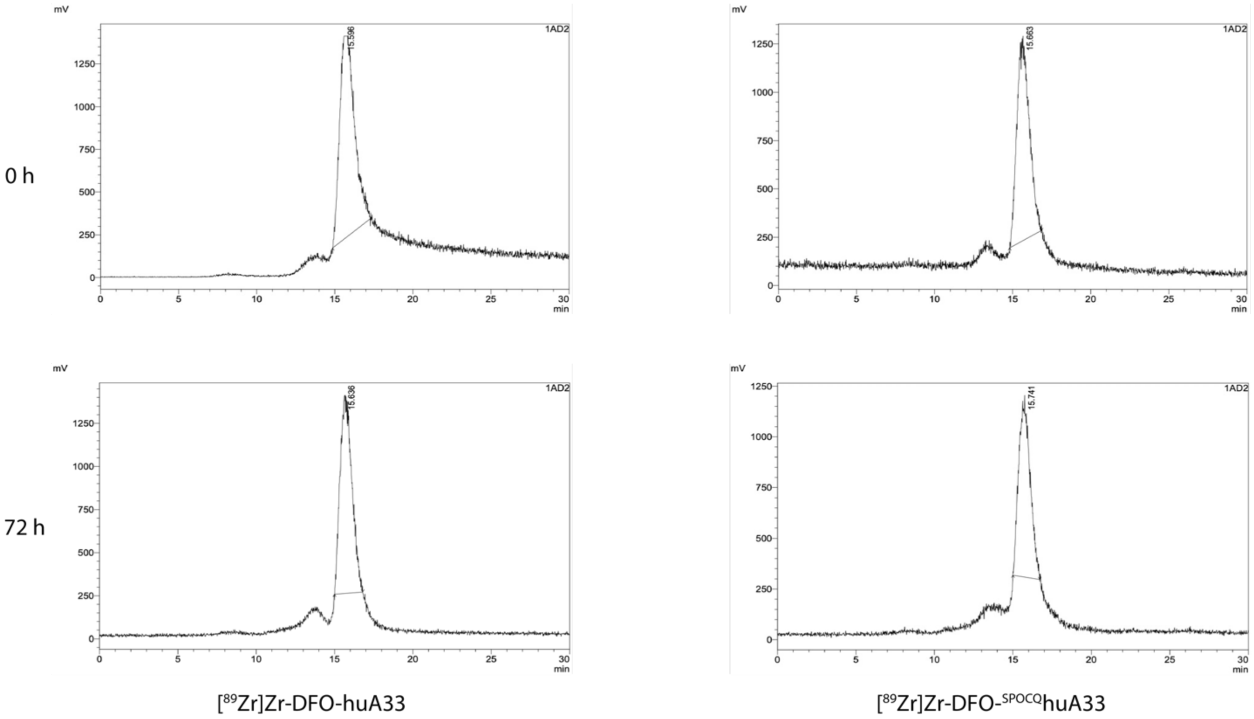

Figure S7. Radio-size-exclusion HPLC for human serum stability study. Following the radiosynthesis of $[^{89}Zr]$Zr-DFO-huA33 and $[^{89}Zr]$Zr-DFO-^{SPOCQ}huA33, the radioimmunoconjugates were incubated in 0.5 mL human serum and placed on a thermomixer at 37 °C, 400 rpm (n = 3). At 0 and 72 h after the start of the study, an aliquot of each radioimmunoconjugate was injected into a size-exclusion HPLC with a radioactivity detector. The peak seen at ~15 minutes is the radiolabeled antibody. Any free $[^{89}Zr]Zr^{4+}$ would be seen as a peak at >20 minutes.

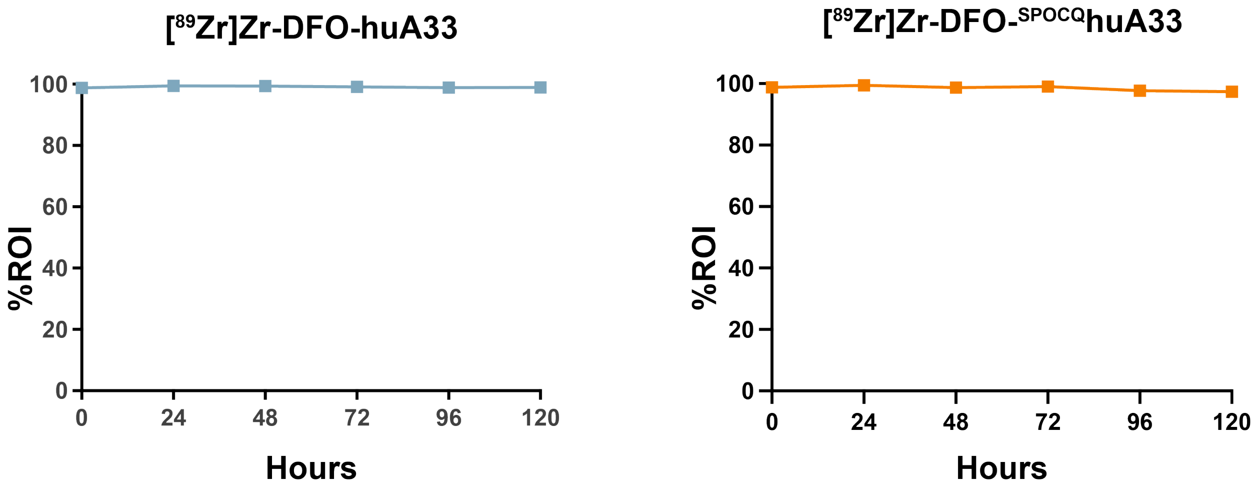

Figure S8. Human serum stability study. Following the radiosynthesis of [^{89}Zr]Zr-DFO-huA33 and [^{89}Zr]Zr-DFO-^{SPOCQ}huA33, the radioimmunoconjugates were incubated in 0.5 mL human serum and placed on a thermomixer at 37 °C, 400 rpm. At 0, 24, 48, 72, 96, and 120 h after the start of the study, each radioimmunoconjugate was assayed via radio-iTLC with EDTA (50 mM, pH 5.0) as an eluent. The % radioactivity associated with the antibody at each timepoint was plotted using GraphPad Prism 8.0 (n = 3). This data corresponds with the data shown in Table S8.

SUPPLEMENTAL TABLES

	**Cy5-^{SPOCQ}huA33 (One-Pot Trial)**	**Cy5-^{SPOCQ}huA33 (High Equivalents)**
**DOL (Cy5/mAb)**	0.2 ± 0.02	1.1 ± 0.1

Table S1. Degree of labeling results of the three-reagent-one-pot approach and the stepwise strategy using a 4 h incubation and quadruple the normal number of equivalents of mTyr and Cy5-TCO.

	**Cy5-^{SPOCQ}trastuzumab**	**Cy5-^{SPOCQ}pertuzumab**
**DOL (Cy5/mAb)**	1.6 ± 0.2	1.7 ± 0.2

Table S2. Degree of labeling results for trastuzumab and pertuzumab site-specifically modified with Cy5 using the mTyr- and the SPOCQ cycloaddition-based strategy.

	DFO-huA33	DFO-^{SPOCQ}huA33
**DOL (DFO/mAb)**	1.7 ± 0.1	1.6 ± 0.1

Table S3. Degree of labeling results for huA33 (a) randomly modified with DFO via the stochastic modification of lysines and (b) site-specifically modified with DFO using our approach based on mTyr and the SPOCQ cycloaddition.

Organs	[^{89}Zr]Zr-DFO-huA33	[^{89}Zr]Zr-DFO-^{SPOCQ}huA33
**Blood**	2.1 ± 1.6	3.0 ± 0.8
**Tumor**	70.0 ± 33.8	109.8 ± 17.6
**Heart**	2.0 ± 0.5	1.5 ± 0.2
**Lungs**	1.4 ± 0.8	1.7 ± 0.2
**Liver**	4.0 ± 1.4	2.7 ± 0.8
**Spleen**	2.0 ± 0.8	2.5 ± 1.1
**Pancreas**	0.7 ± 0.3	0.6 ± 0.2
**Stomach**	0.6 ± 0.2	0.5 ± 0.2
**S. Intestine**	1.2 ± 0.4	0.6 ± 0.1
**L. Intestine**	0.9 ± 0.0	0.5 ± 0.1
**Kidneys**	1.7 ± 0.8	4.9 ± 0.5
**Ovaries**	1.4 ± 0.6	1.2 ± 1.1
**Muscle**	0.4 ± 0.3	0.5 ± 0.2
**Bone**	4.6 ± 0.5	7.0 ± 5.3
**Skin**	2.1 ± 0.9	1.8 ± 1.2
**Tail**	3.1 ± 2.3	1.5 ± 0.1

Table S4. **Biodistribution data from mice bearing SW1222 xenografts.** Biodistribution data collected 120 h after the intravenous administration of [^{89}Z]Zr-DFO-huA33 or [^{89}Zr]Zr-DFO-^{SPOCQ}huA33 [3.7 -4.0 MBq (100-110 μCi), 20-22 μg, in 100 μL of PBS] to athymic nude mice bearing A33-expressing SW1222 colorectal carcinoma xenografts (n = 4). Values are in units of %ID/g and are expressed as mean ± standard deviation.

Organs	[^{89}Zr]Zr-DFO-huA33	[^{89}Zr]Zr-DFO-^{SPOCQ}huA33
**Blood**	0.3 ± 0.1	5.7 ± 0.4
**Tumor**	33.1 ± 10.6	76.2 ± 13.0
**Heart**	1.8 ± 0.6	1.1 ± 0.6
**Lungs**	1.6 ± 0.2	3.3 ± 1.8
**Liver**	16.2 ± 0.6	5.9 ± 2.1
**Spleen**	42.1 ± 8.6	9.7 ± 4.0
**Pancreas**	1.1 ± 0.8	0.9 ± 0.6
**Stomach**	1.1 ± 0.4	1.2 ± 0.1
**S. Intestine**	5.1 ± 0.4	1.7 ± 0.2
**L. Intestine**	1.2 ± 0.6	2.9 ± 3.7
**Kidneys**	1.4 ± 0.3	6.2 ± 5.7
**Ovaries**	3.1 ± 1.4	1.0 ± 0.2
**Muscle**	0.3 ± 0.1	0.3 ± 0.1
**Bone**	8.2 ± 5.1	6.6 ± 0.5
**Skin**	2.2 ± 0.2	3.8 ± 0.4
**Tail**	2.7 ± 1.3	2.0 ± 0.2

Table S5. **Biodistribution data from mice bearing SW1222 xenografts.** Biodistribution data collected 120 h after the intravenous administration of [^{89}Z]Zr-DFO-huA33 or [^{89}Zr]Zr-DFO-^{SPOCQ}huA33 [3.7 -4.0 MBq (100-110 μCi), 20-22 μg, in 100 μL of PBS] to NSG mice bearing A33-expressing SW1222 colorectal carcinoma xenografts (n = 4). Values are in units of %ID/g and are expressed as mean ± standard deviation.

	Absorbance @ 450 nm			
**Immunoconjugate Concentration (μg/ml)**	**huA33**	**^{degly}huA33**	**DFO-huA33**	**DFO-^{SPOCQ}huA33**
**1000**	0.44 ± 0.03	0.45 ± 0.01	0.41 ± 0.02	0.43 ± 0.004
**333.3**	0.41 ± 0.02	0.44 ± 0.01	0.38 ± 0.02	0.41 ± 0.01
**111.1**	0.38 ± 0.31	0.41 ± 0.002	0.34 ± 0.02	0.38 ± 0.01
**37.03**	0.31 ± 0.01	0.35 ± 0.01	0.26 ± 0.02	0.33 ± 0.003
**12.34**	0.2 ± 0.01	0.25 ± 0.01	0.17 ± 0.002	0.25 ± 0.004
**4.115**	0.12 ± 0.01	0.16 ± 0.02	0.10 ± 0.001	0.18 ± 0.01
**1.371**	0.07 ± 0.002	0.11 ± 0.01	0.07 ± 0.003	0.15 ± 0.03
**0.475**	0.06 ± 0.002	0.10 ± 0.02	0.06 ± 0.01	0.14 ± 0.01

Table S6. ELISA results for A33 binding. A33-coated plates were incubated with varying concentrations of huA33, ^{degly}huA33, DFO-huA33, or DFO-^{SPOCQ}huA33 (n = 3). After a 2 h incubation, HRP-labeled goat anti-human secondary antibody was incubated in each well for 1 h followed by TMB for 10 minutes. The absorbance of each well was detected on a microplate reader, and the results were analyzed using GraphPad Prism 8.0 software. This data corresponds with the data shown in Figure 2.

	**Absorbance @ 450 nm**			
**Immunoconjugate Concentration (µg/ml)**	**huA33**	**^{degly}huA33**	**DFO-huA33**	**DFO-^{SPOCQ}huA33**
**1000**	0.49 ± 0.01	0.14 ± 0.01	0.46 ± 0.01	0.18 ± 0.01
**333.3**	0.44 ± 0.01	0.10 ± 0.002	0.41 ± 0.01	0.11 ± 0.002
**111.1**	0.38 ± 0.01	0.07 ± 0.00	0.35 ± 0.01	0.07 ± 0.001
**37.03**	0.28 ± 0.01	0.05 ± 0.003	0.24 ± 0.01	0.06 ± 0.002
**12.34**	0.16 ± 0.002	0.04 ± 0.001	0.13 ± 0.004	0.05 ± 0.001
**4.115**	0.09 ± 0.002	0.04 ± 0.00	0.08 ± 0.003	0.05 ± 0.003
**1.371**	0.06 ± 0.001	0.04 ± 0.001	0.06 ± 0.004	0.05 ± 0.01
**0.475**	0.07 ± 0.003	0.04 ± 0.001	0.05 ± 0.001	0.04 ± 0.001

Table S7. ELISA results for hFcγRI binding. hFcγRI- coated plates were incubated with varying concentrations of huA33, ^{degly}huA33, DFO-huA33, or DFO-^{SPOCQ}huA33 (n = 3). After a 2 h incubation, HRP-labeled goat anti-human secondary antibody was incubated in each well for 1 h followed by TMB for 10 minutes. The absorbance of each well was detected on a microplate reader, and the results were analyzed using GraphPad Prism 8.0 software. This data corresponds with the data shown in Figure 2.

	%ROI of radio-iTLC					
**Constructs**	**0 h**	**24 h**	**48 h**	**72 h**	**96 h**	**120 h**
**[^{89}Zr]Zr-DFO-huA33**	98.8 ± 0.3	99.4 ± 0.2	99.4 ± 0.2	99.1 ± 0.2	98.9 ± 0.1	99.0 ± 0.2
**[^{89}Zr]Zr-DFO-^{SPOCQ}huA33**	98.8 ± 0.2	99.4 ± 0.1	98.7 ± 0.3	99.0 ± 0.0	97.7 ± 0.3	97.4 ± 0.6

Table S8. Human serum stability study results. Following the radiosynthesis of [^{89}Zr]Zr-DFO-huA33 and [^{89}Zr]Zr-DFO-^{SPOCQ}huA33, the radioimmunoconjugates were incubated in 0.5 mL human serum and placed on a thermomixer at 37 °C, 400 rpm. At 0, 24, 48, 72, 96, and 120 h after the start of the study, each radioimmunoconjugate was assayed via radio-iTLC using EDTA (50 mM, pH 5.0) as the eluent (n = 3). This data corresponds with the data shown in Figure S8.
